# Isolated Anterior Mesenteric Neurofibroma: A Rare Manifestation of Neurofibromatosis Type 1

**DOI:** 10.7759/cureus.102836

**Published:** 2026-02-02

**Authors:** Sara Ouassil, Mariem Touraif, Soumia Mrhar, Najoua Aballa, Isaac Mpanya Ntumba, Hussein Choukri Ahmanna, Ibtissam Zouita, Dounia Basraoui, Salma Foura, Mohamed Oulad Saiad, Mohammed Bouskraoui, Hicham Jalal

**Affiliations:** 1 Mother and Child Department, Radiology, University Hospital Center Mohammed VI, Faculty of Medicine and Pharmacy, Cadi Ayyad University, Marrakech, MAR; 2 Mother and Child Department, Pediatric Surgery, University Hospital Center Mohammed VI, Faculty of Medicine and Pharmacy, Cadi Ayyad University, Marrakech, MAR; 3 Mother and Child Department, General Pediatrics, University Hospital Center Mohammed VI, Faculty of Medicine and Pharmacy, Cadi Ayyad University, Marrakech, MAR

**Keywords:** child, gastrointestinal involvement, imaging, mesentery, neurofibroma

## Abstract

Neurofibromatosis type 1 (NF1), also known as Von Recklinghausen disease, is a multisystemic, hereditary, autosomal dominant condition. It is caused by the development of tumors in the nervous system, resulting from mutations in the *NF1* gene located on chromosome 17q11.2. Gastrointestinal involvement is mainly extraperitoneal, with mesentery lesions being the least common. We report a case of an isolated jejunal mesenteric neurofibroma diagnosed by ultrasound and CT scan and confirmed by histopathological examination in a nine-year-old child with diffuse café-au-lait spots who presented with chronic abdominal pain.

## Introduction

Neurofibromatosis type 1 (NF1) is an autosomal dominant disorder affecting approximately 1 in 3,000 births [1]. This condition primarily involves the integumentary system and peripheral nerves and is essentially defined by the occurrence of café-au-lait macules and neurofibromatous lesions distributed along peripheral nerve pathways [2].

Patients commonly present with single or multiple neurofibromas, including plexiform variants, as well as leiomyomas and other associated neoplasms [3]. Gastrointestinal manifestations are observed in approximately 10-25% of affected individuals and may consist of isolated or multiple neurofibromas, leiomyomas, and, less frequently, plexiform neurofibromas [4].

Involvement of the colon and mesentery is uncommon. Because mesenteric disease is often clinically silent, diagnosis may be delayed, potentially resulting in increased morbidity [5,6].

In this paper, we report a case of an isolated anterior mesenteric neurofibroma in a nine-year-old child. Furthermore, we provide a review of the pediatric literature focusing on clinical manifestations, diagnostic strategies, and management of comparable cases.

## Case presentation

**Figure 1:**
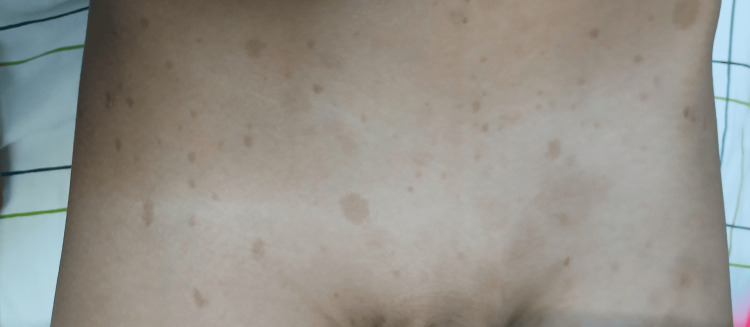
Diffuse café-au-lait macules

We report the case of a nine-year-old child with a known diagnosis of NF1, established based on two major diagnostic criteria: a first-degree family history of NF1 (father under medical follow-up) and the presence of multiple café-au-lait macules. The patient presented with chronic abdominal pain that had progressively worsened over the past six months, associated with recurrent episodes of vomiting. Physical examination revealed a child in good general condition, with multiple café-au-lait macules (Figure 1). Abdominal palpation identified a palpable mass measuring approximately 9 cm, associated with mild abdominal tenderness, predominantly localized in the pelvic region.

**Figure 2:**
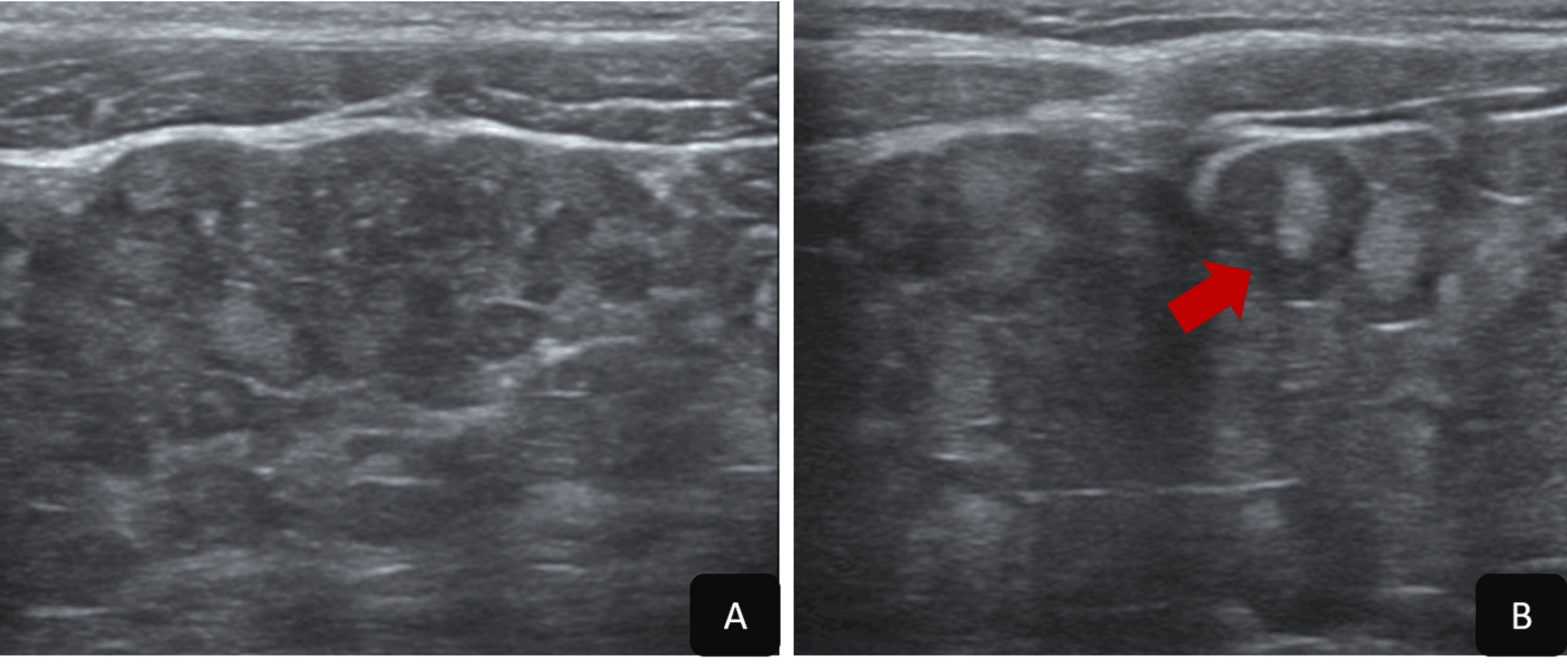
Abdominal ultrasound (A, B) Anterior mesenteric mass, relatively well-defined with lobulated contours, containing nodular structures with a hyperechoic center and a peripheral hypoechoic ring, producing a characteristic 'target' appearance (red arrow).

Abdominal ultrasound (US) revealed the presence of an anterior mesenteric mass, relatively well-defined with lobulated contours, containing nodular structures with a hyperechoic center and a peripheral hypoechoic ring, producing a characteristic 'target' appearance (Figure 2). No vascularization was detected on color Doppler imaging (Figure 2).

**Figure 3:**
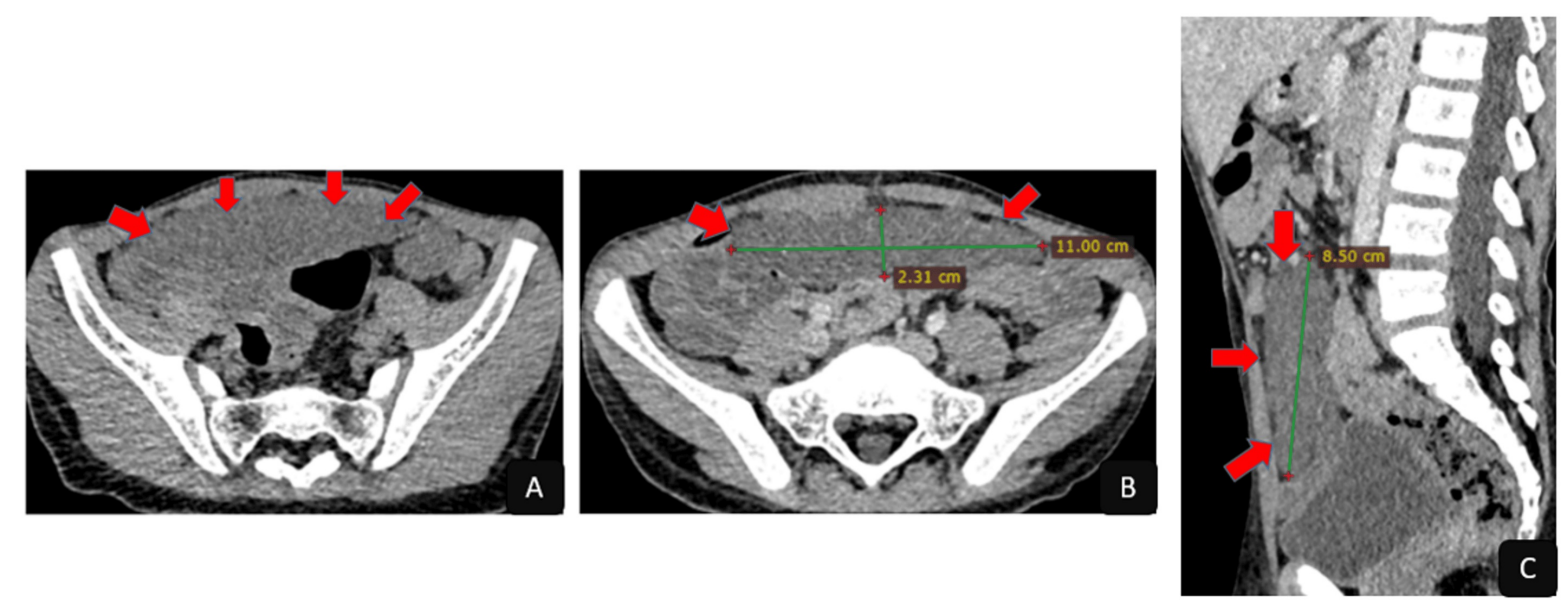
Abdominopelvic CT scan (A) Axial slice before contrast administration; (B) axial slice after contrast administration; (C) sagittal reconstruction. Well-defined, spontaneously hypodense anterior mesenteric mass, with no enhancement following contrast administration.

**Figure 4:**
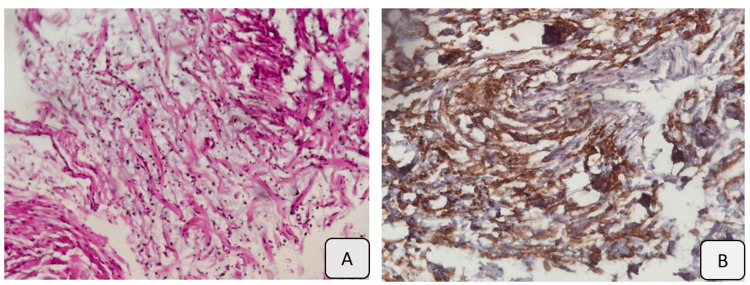
Histopathological results (A) Hematoxylin and eosin (H&E) staining; (B) immunohistochemical (IHC) analysis. A low-density spindle cell population with moderate immunoreactivity of tumor cells for anti-CD34 antibodies.

Computed tomography (CT) confirmed the anatomical location and solitary nature of the lesion, demonstrating a well-circumscribed anterior mesenteric mass with spontaneous hypodensity (26 Hounsfield units). The lesion measured 110 × 23 × 85 mm (transverse × anteroposterior × craniocaudal) and showed no significant enhancement after contrast administration (30 Hounsfield units). Thin venous vascular structures were observed coursing through the lesion (Figure 3). An ultrasound-guided biopsy was performed, confirming the diagnosis of mesenteric neurofibroma (Figure 4).

**Figure 5:**
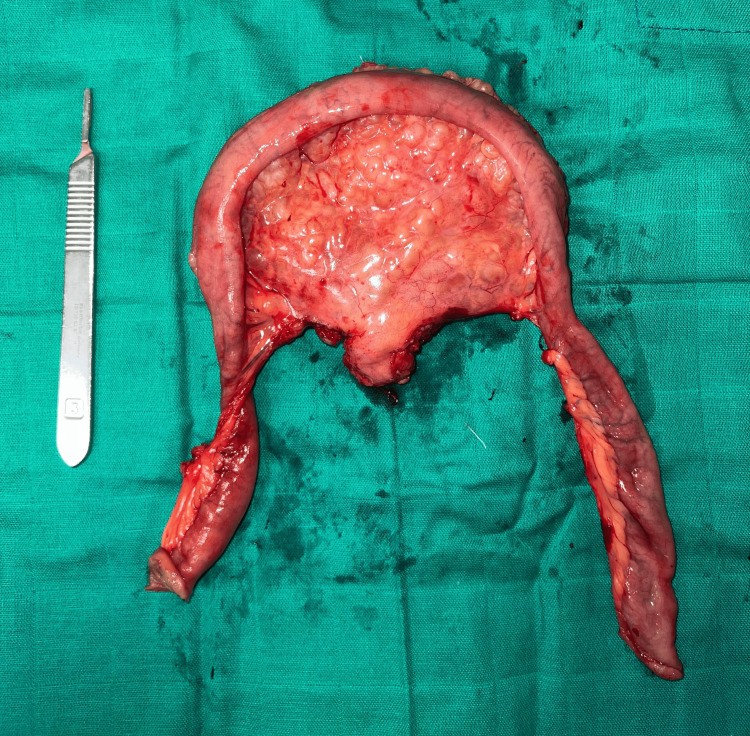
Image of the surgical specimen showing the resected mass arising from the jejunal mesentery, with approximately 40 cm of small intestine resected

The patient subsequently underwent surgical exploration, first laparoscopically, then converted to a minimal laparotomy, allowing the resection of the mass (Figure 5). The mass was located in the mid-jejunal mesentery. The bowel was spared, and no communication with the lumen was identified macroscopically during the procedure. The bowel resection was indicated due to the compromise of the bowel vascular irrigation. The clinical course was marked by a complete resolution of symptoms, with no evidence of recurrence on abdominal US performed three months after the surgical procedure.

## Discussion

NF1 is a multisystem disorder caused by defects in the *NF1* gene, leading to abnormal development of neural crest-derived cells [7]. It is an autosomal dominant condition classified among cutaneous neurocutaneous syndromes, with frequent involvement of the skin, skeletal system, trunk, and peripheral nerves [8]. Gastrointestinal involvement affects up to one-quarter of patients [9,10]. The upper gastrointestinal tract is most frequently affected, with neurofibromas representing the predominant lesion type, most commonly arising in the small intestine [11]. However, the esophagus and colon are rarely involved [12].

Mesenteric neurofibromatosis is a very uncommon NF1 manifestation [13]. The clinical spectrum ranges from asymptomatic cases to a broad array of gastrointestinal manifestations, including abdominal pain, weight loss, diarrhea, mucosal ulceration, bowel obstruction, intussusception, and volvulus [14]. However, 65% of patients with small mesenteric neurofibromatosis are asymptomatic [14]. Authors reporting mesenteric involvement in pediatric patients with NF1 have noted predominantly vague and non-specific clinical symptoms, such as abdominal pain, abdominal distension, and vomiting (Table 1).

**Table 1:** Related studies of mesenteric neurofibromas associated with NF1 in children

Study	Age	Gender	Symptoms	Involved mesentery	Surgery	Medical treatment
Imamoğlu et al. [4]	11	F	Abdominal pain and failure to thrive	Panmesenteric	Surgical biopsy without total resection	Symptomatic
Sang et al. [13]	5	M	Abdominal distention and intermittent abdominal pain	Diffuse	Surgical biopsy without total resection	Not mentioned
Gorbounova [14]	16	F	Faltering growth, abdominal pain, and chronic diarrhea	Extensive	Surgical biopsy without total resection	Symptomatic
Matsuki et al. [20]	10	M	Abdominal pain, nausea, and vomiting	Distal ileum	Total resection	-
Kataria et al. [21]	6	M	None	Ileum	Total resection	-
Kandhakatla [22]	15	M	Abdominal pain, vomiting, and abdominal distension	Ileum	Total resection	-
Present case	9	M	Chronic abdominal pain with recurrent episodes of vomiting	Jejunal mesentery	Total resection of the mass	Not required

On imaging, mesenteric neurofibromatosis typically presents as multiple distinct nodules or as infiltrative lesions extending from the mesenteric root to the intestinal wall [15]. In the literature, mesenteric involvement in the setting of NF1 is most often diffuse, with predominant involvement of the ileum (Table 1). Hence, the distinctive feature of our case is that it involves an isolated and well-defined jejunal mesenteric neurofibroma.

On ultrasound, these lesions appear hypoechoic, exhibiting either a homogeneous or heterogeneous internal echotexture, depending on their size [16]. This imaging appearance may mimic other common pediatric conditions, particularly lymphoma, which is frequently associated with gastrointestinal wall thickening and hepatosplenomegaly; inflammatory myofibroblastic tumors, characterized by variable echogenicity; as well as desmoid tumors, although these remain rare in the pediatric population.

CT imaging typically demonstrates homogeneously low-attenuation masses on post-contrast studies [6]. These features are thought to result from entrapped adipose tissue, cystic degeneration, and the presence of a myxoid matrix. Less frequently, the lesions may exhibit calcifications or post-contrast enhancement [6].

On magnetic resonance imaging (MRI), these lesions typically demonstrate low signal intensity on T1-weighted images and variable signal intensity on T2-weighted images, with cystic or myxoid regions appearing hyperintense and collagenous or fibrotic areas appearing hypointense, which enhance following gadolinium administration [17]. A characteristic "target sign" may be observed on T2-weighted images, showing central hypointensity surrounded by a hyperintense rim, while T1-weighted images remain hypointense. Post-contrast imaging generally reveals mild to moderate enhancement [16].

Positron emission tomography (PET) scans are useful in detecting signs of malignant transformation in neurofibromas. Caution should be exercised when interpreting fludeoxyglucose (FDG) uptake in neurofibromas, as benign lesions may also exhibit a wide range of FDG avidity on PET imaging. Thus, elevated FDG uptake in neurofibromas does not necessarily signify malignancy and may instead reflect areas of dense collagen and increased cellularity [18].

Histologically, neurofibromas are composed of Schwann cells, fibroblasts, and a myxoid or mucinous matrix, all embedded within collagenous tissue and often containing mast cells and adipocytes, and may be associated with cystic degeneration. They may appear as solitary, well-circumscribed masses or as plexiform lesions [13]. Malignant transformation has been reported in 5-15% of cases, particularly in patients older than 40 years [11].

Management begins with a biopsy to rule out possible malignancy [2]. Surgical intervention has traditionally been recommended for symptomatic or rapidly enlarging neurofibromas, when technically feasible, to alleviate pain, bleeding, obstruction, and other mass-effect-related symptoms [19]. However, in cases in which the mass is unresectable, the optimal management consists of biopsy to exclude malignancy, followed by oral thalidomide, an anti-angiogenic agent, with close tumor follow-up [6]. In the cases reported in the literature, complete surgical resection was performed whenever feasible; otherwise, partial surgical resection was undertaken (Table 1). Recently, selective small-molecule inhibitors of mitogen-activated protein kinase (MEK1/2) have shown effectiveness in decreasing tumor volume and alleviating mass-effect-related symptoms in pediatric patients with plexiform neurofibromas [19].

## Conclusions

Mesenteric neurofibromatosis is a rare condition, particularly in its well-circumscribed form. Imaging plays a crucial role in lesion detection and characterization, while the diagnosis is confirmed histologically. Timely diagnosis is crucial to enable curative management before the mass reaches an unresectable size. Ideally, optimal management of resectable mesenteric neurofibroma involves performing a biopsy to exclude malignancy, followed by surgical resection to prevent involvement of vital structures. However, particularly in cases of unresectable neurofibromas, medical management plays a pivotal role, especially since the advent of novel therapeutic agents, namely selective small-molecule inhibitors of MEK1/2, which have demonstrated clinical efficacy in the pediatric population.
